# Towards Creating the Perfect* In Vitro* Cell Model

**DOI:** 10.1155/2016/3459730

**Published:** 2016-02-03

**Authors:** Malin K. B. Jonsson, Toon A. B. van Veen, Jane Synnergren, Bruno Becker

**Affiliations:** ^1^Genome Institute of Singapore, 60 Biopolis Street, Genome, Singapore 138672; ^2^Department of Medical Physiology, Division of Heart & Lungs, UMC Utrecht, 3584 CM Utrecht, Netherlands; ^3^Systems Biology Research Center, University of Skövde, 541 28 Skövde, Sweden; ^4^Department of Psychiatry and Neurochemistry, Sahlgrenska University Hospital, 431 80 Mölndal, Sweden

Researchers both in academia and in industry regularly rely on* in vitro* models to study biological responses and mechanisms related to human health and disease. These models range from relatively simple overexpression systems in, for example, HEK293 or COS cells to more complex specialized cells/tissues/organs from animals or, in rare cases, humans (e.g., skin models, explanted organ materials) [1, 2]. Even though the results from these* in vitro* studies are further tested and validated in* in vivo* animal models before being extrapolated to the human situation, in general the translation of results from bench to bedside remains challenging due to poor species overlap. This is contributing to the high attrition rate that the pharmaceutical industry has been struggling with over the past years when trying to introduce new drugs into the market [3]. Nevertheless,* in vitro* models are one of the pillars of contemporary research, with the benefits of ease of pharmacological manipulation, genetic modifications, and analysis, small sample sizes, and relatively low cost. Although a complete abolishment of animal models is highly unlikely in the near future, the importance of cell models will increase as initiatives like the 3Rs (refining, reducing, and replacing animal models) are globally gaining importance. The US FDA is even planning to adapt its guidelines for preclinical cardiotoxicity studies by incorporating a computational integration of various individual ion channel assays as well as electrophysiological tests on stem cell-derived cardiomyocytes [4]. Needless to say, it stresses that a continuous improvement of our* in vitro* models is therefore warranted.

One of the reasons for the mentioned high attrition rates in the pharmaceutical industry is the lack of sufficiently predictive preclinical models of human origin. The discovery of human (induced) pluripotent stem cells (hPSC) and their ability to differentiate into a large variety of different cell types [5, 6] has therefore raised the hope for being able to create increasingly reliable* in vitro* models which includes the possibility of further exploring their potential in the field of personalized medicine. Extensive evaluation of these models has indeed shown great promise. For example, hPSC-derived hepatocytes that were exposed to hepatotoxic compounds over a long period of time (14 days) showed evidence of phospholipidosis and steatosis, which are signs of chronic long term toxicity [7]. Yet another example is the generation of functional hippocampal neurons from hPSC and their usefulness for studies on the onset and progression of diseases such as Alzheimer's disease and epilepsy [8]. Taking it one step further, researchers have also been able to test novel therapeutic options for the treatment of autosomal-dominant-negative disorders. This was elegantly shown through the use of RNA interference (RNAi) that rescued the diseased phenotype of hPSC-derived cardiomyocytes carrying a mutation causing the long QT syndrome [9] or carrying a mutation in phospholamban that results either in dilated or in arrhythmogenic cardiomyopathy [10].

Despite all these promising results, there are still severe limitations that currently hinder the implementation of stem cell models in routine assays. One of the most important and general challenges related to stem cell-derived models is the immature or somewhat artificial phenotype of the derived cells which consequently leaves a considerable gap to the* in vivo* situation. This seems to hold true for all specialized cell types that are derived from hPSC and can be exemplified by, for example, hPSC-derived hepatocytes that persistently express fetal markers like alpha fetoprotein (AFP) and also lack key mature hepatocyte functions, as reflected by low levels of many detoxification enzymes (e.g., CYP2A6, CYP3A4) [11]. Another remaining challenge is to efficiently, and across different hPSC lines, control the directed differentiation into a specific subtype of a particular lineage, such as GABAergic cortical interneurons or pancreatic *β*-cells [12, 13].

For this special issue, investigators have contributed original research articles as well as review articles that will support the research community in approaching the goal of obtaining models for the lab that can mirror the* in vivo* situation. The published manuscripts in this special issue make use of stem cells to derive hepatocytes, cartilage, and pancreatic cells as well as utilize mesenchymal and hematopoietic stem cells under different conditions. Various methodologies have been employed to improve the cell phenotype generated, for example, by modulating of substrate stiffness, creating of functional scaffolds, and the addition of drugs. Furthermore, a range of topics, including cell cycle senescence, DNA methylation, and the use of CRISPR-Cas9 for genome editing, are covered. We hope that the readers will appreciate the contents of this issue and find scientific inspiration and new ideas for their future work.

*Malin K. B. Jonsson* *Malin K. B. Jonsson* *Toon A. B. van Veen* *Toon A. B. van Veen* *Jane Synnergren* *Jane Synnergren* *Bruno Becker* *Bruno Becker*
